# Anti-saccades predict cognitive functions in older adults and patients with Parkinson’s disease

**DOI:** 10.1371/journal.pone.0207589

**Published:** 2018-11-28

**Authors:** Julie Ouerfelli-Ethier, Basma Elsaeid, Julie Desgroseilliers, Douglas P. Munoz, Gunnar Blohm, Aarlenne Zein Khan

**Affiliations:** 1 School of Optometry, University of Montreal, Montreal, Quebec, Canada; 2 Centre for Neuroscience Studies, Queen’s University, Kingston, Ontario, Canada; 3 Department of Medicine and Health Sciences, University of Sherbrooke, Sherbrooke, Quebec, Canada; Nathan S Kline Institute, UNITED STATES

## Abstract

A major component of cognitive control is the ability to act flexibly in the environment by either behaving automatically or inhibiting an automatic behaviour. The interleaved pro/anti-saccade task measures cognitive control because the task relies on one’s abilities to switch flexibly between pro and anti-saccades, and inhibit automatic saccades during anti-saccade trials. Decline in cognitive control occurs during aging or neurological illnesses such as Parkinson’s disease (PD), and indicates decline in other cognitive abilities, such as memory. However, little is known about the relationship between cognitive control and other cognitive processes. Here we investigated whether anti-saccade performance can predict decision-making, visual memory, and pop-out and serial visual search performance. We tested 34 younger adults, 22 older adults, and 20 PD patients on four tasks: an interleaved pro/anti-saccade, a spatial visual memory, a decision-making and two types of visual search (pop-out and serial) tasks. Anti-saccade performance was a good predictor of decision-making and visual memory abilities for both older adults and PD patients, while it predicted visual search performance to a larger extent in PD patients. Our results thus demonstrate the suitability of the interleaved pro/anti-saccade task as a cognitive marker of cognitive control in aging and PD populations.

## Introduction

While Parkinson’s disease (PD) is primarily understood as a motor disorder, non-motor symptoms such as cognitive impairment are present from the early stages of the disorder [1]. PD patients’ cognitive deficits comprise a wide range of abilities including selective attention [2], response inhibition [3], visual-spatial processing [4], memory [5], task-switching [6], decision-making [7], and the planning of goal-directed behaviours [8,9]. It was hypothesized that the underlying cause of PD cognitive impairment is a deficit in inhibitory control, a key component in many cognitive abilities and intrinsic to cognitive control [6]. Cognitive control is the ability to act flexibly, or alternate between inhibiting unwanted behaviours in favour of goal-directed ones, and responding automatically. In other words, it involves both inhibitory control and task-switching abilities [10]. These abilities are required in performing standardized neuropsychological tasks such as the Stroop task where one must actively ignore the colour of the letters and instead read the name of a colour [11]. Indeed, PD patients experience difficulty during the Stroop task because of deficits in cognitive control; they have impaired inhibitory control and task-switching abilities [12,13].

Cognitive control can also be measured by non-standardized tests using the oculomotor system, thus offering a faster and simpler alternative to standardized testing. For instance, the interleaved pro/anti-saccade task requires task-switching and inhibitory control abilities and can, as a result, measure cognitive control in various clinical populations, notably in PD [3,14–16]. The task requires participants to alternate between two types of rapid eye-movements: pro- and anti-saccades [17]. Pro-saccades are an automatic-type of eye-movement, where a novel stimulus in the visual field attracts the gaze. Anti-saccades, on the contrary, rely on two sub-processes: a) inhibiting an automatic-type of eye-movement towards a novel stimulus, and b) generating a voluntary movement in exactly the opposite direction from the novel stimulus, where nothing in the visual field is attracting the gaze. The interleaved pro- and anti-saccade task requires participants to alternate between automatic (i.e., pro-saccades) and voluntary behaviours (i.e., anti-saccades), thus necessitating cognitive control abilities [18]. Indeed, this paradigm reveals an impairment in switching costs and more frequent pro-saccades during anti-saccades trials in PD [6,19–21].

Anti-saccade performance is also correlated with results in neuropsychological tests in healthy adults [22], in older adults [23], and in PD patients’ performance in the task correlates with mild cognitive impairment and dementia [24]. Additionally, previous research has shown that anti-saccade performance was significantly correlated with cognitive deficits revealed by tests such as the Wisconsin Card Sort Test, which evaluates cognitive control [25,26].

Cognitive control abilities and saccades recruit the dorsolateral prefrontal cortex [27], known to be involved in executive functioning [28]. In the case of PD, neural degeneration occurs in the basal ganglia [29,30] which affects the frontal lobe through pathways linking the basal ganglia and frontal lobes. Further, these striato-frontal pathways are associated with inhibitory control abilities [31,32] and other cognitive processes such as memory [5,33]. These overlapping neuronal circuits suggest common functional processes between cognitive control and various cognitive functions [6]. Recent findings in PD also imply the sensitivity of anti-saccades in certain task paradigms could assess cognitive decline and DLPC dysfunction [34]. Yet, whether anti-saccades measure cognitive control involved in performing cognitive tasks remains largely unexplored in aging populations. Our goal is to investigate the potential of the interleaved pro/anti-saccade task as a sensitive indirect measure (or predictor) of cognitive functioning in aging or a neuropathology such as PD. Identifying these relationships at an early stage would be crucial in the clinical assessment of PD; there is currently no official screening test for cognitive impairment [35].

Here we compared three groups, younger healthy adults, PD patients in the early stages of the disease, and age-matched controls, to evaluate the relationships between age, pathology, and cognitive functioning. We correlated performance in the interleaved pro- and anti-saccade task with a series of visual cognitive tasks testing decision-making abilities [36], and bottom-up and top-down visual search [37,38], and spatial visual memory [39].

## Methods and materials

### Participants

We recruited a total of 76 participants from the community and via the Parkinson Quebec Network (Montreal, QC): 34 younger adults (*M* = 22.7 y, *SD* = 3.7, age range: 19–37 y), 22 older adults (*M*_age_ = 65.6 y, *SD*_age_ = 8.2, age range: 52–83 y), and 20 medicated patients with mild to moderate idiopathic PD (*M*_age_ = 67.4 y, *SD*_age_ = 8.3, age range: 52–85 y; see Table 1 for Hoehn and Yahr staging, medication information and demographics). Older adults were age-matched to PD patients, within 2 years.

**Table 1:** Patient demographics and clinical measures.

Patients	Sex	Age (y)	Education (y)	H&Y	Medications	
					Generic name	Dosage (mg)
1	M	77	21	1.5	L	100/25
2	F	69	13.5	2.5	Se	5
3	M	69	18	2	.	.
4	M	85	18	≤ 3	L	100/25
5	M	59	16	1	L, Sa	100/25, 30
6	F	69	16	2	L	100/25
7	M	54	14	2	L	100/25
8	F	62	13	2	L	100/25
9	M	54	17	≤ 3	L	150
10	M	69	16	≤ 3	L	100/25
11	F	53	16	1.5	L, R	100/25, 0.5
12	M	74	6	2	L	100/50
13	M	64	12	1	L	100/25
14	F	67	21	3	L	100/25
15	M	74	10	2	L	100/25
16	M	66	14	2	L, Sa	100/25, 0.4
17	F	66	14.5	1	L	100/25
18	M	68	13	1	L	100/25
19	M	70	13	1	L	100/25
20	F	79	16	1.5	L	100/25

H&Y, Hoehn and Yahr Scale; L, Levodopa/carbidopa/entacapone; R, Rasagiline; Sa, Sandoz; Se, Selegiline. Missing data are illustrated by a dot.

We screened older and PD participants for moderate or severe cognitive impairment, or dementia, using the Montreal Cognitive Assessment (MoCA) [40] and Mini-Mental State Examination (MMSE) [41]. We included participants with mild cognitive impairment with a cut-off point of 23 (see Table 2 for participants’ mean MoCA and MMSE scores) [42]. This is aligned with previous findings reporting the ability to give informed consent for PD patients is above a score of 22 on the MoCA [43].

**Table 2:** MoCA and MMSE score differences between older and PD cohorts.

Variable	*n*	*M*	*SD*	*F*	*p*
MoCA				8.66	< .01
Older adults	22	27.8	2.3		
PD patients	20	26	2		
MMSE				2.40	.13
Older adults	22	28.3	1.7		
PD patients	20	29.1	1.4		

n, subsample size; M, mean; SD, standard-deviation; F, one-way ANOVA with group as a factor; MoCA, Montreal Cognitive Assessment; MMSE, Mini-Mental State Examination.

All participants had normal or corrected-to-normal vision. Participants completed a general health questionnaire and those with any other neurological or psychiatric disorders were excluded. Patient’s medication intake and dosage were not controlled for ethical reasons. The experimental design was approved by the ethical committees at Queen’s University and at the University of Montreal. Written informed consent was obtained from all participants before testing, and they received financial compensation for their participation upon completion. One younger participant did not complete the decision-making task. Two participants did not complete the pop-out visual search task: one PD patient and one young adult. As a result, they were only included in analyses for the serial search task.

### Materials and procedure

Participants performed five cognitive tasks in randomized order while their eye movements were recorded: interleaved pro/anti saccade, spatial visual memory, decision-making, pop-out visual search, and serial visual search during one session of approximately 1h30. Each task was blocked with few trials per block in order to encourage breaks and prevent fatigue in participants. The number of blocks and trials per task are described below.

All tasks were designed and implemented using MatLab (The MathWorks, Inc., Natick, Massachusetts, United States) with Psychophysics toolbox [44]. Testing occurred at Queen’s University (Kingston, Canada) and at the University of Montreal (Montreal, Canada) with almost identical setups. Participants sat in a dark room 60 cm away from computer screen (at Queens: 20” Mitsubishi Diamond Pro CRT, 16x12 inches, 1280x1024 pixels, 60Hz, at the University of Montreal: VIEWpixx 3D (VPixx Technologies, Montreal, Canada) 20.5*11.5 inches, 1920*1080 pixels, 120 Hz). An EyeLink 1000 Plus eye-tracker set in a binocular tower mount (SR Research, Kanata, Canada) recorded eye movements at 1000 Hz. During recordings, participants’ head movements were restricted with chin- and forehead-rests. Button-press responses to spatial visual memory, decision-making, and visual search tasks were recorded using a response box (at Queens: SR Research Gamepad (SR Research, Kanata, Canada), at the University of Montreal: RESPONSEpixx handheld response box (VPixx Technologies, Montreal, Canada).

#### Interleaved pro- anti-saccade task

**Fig 1:**
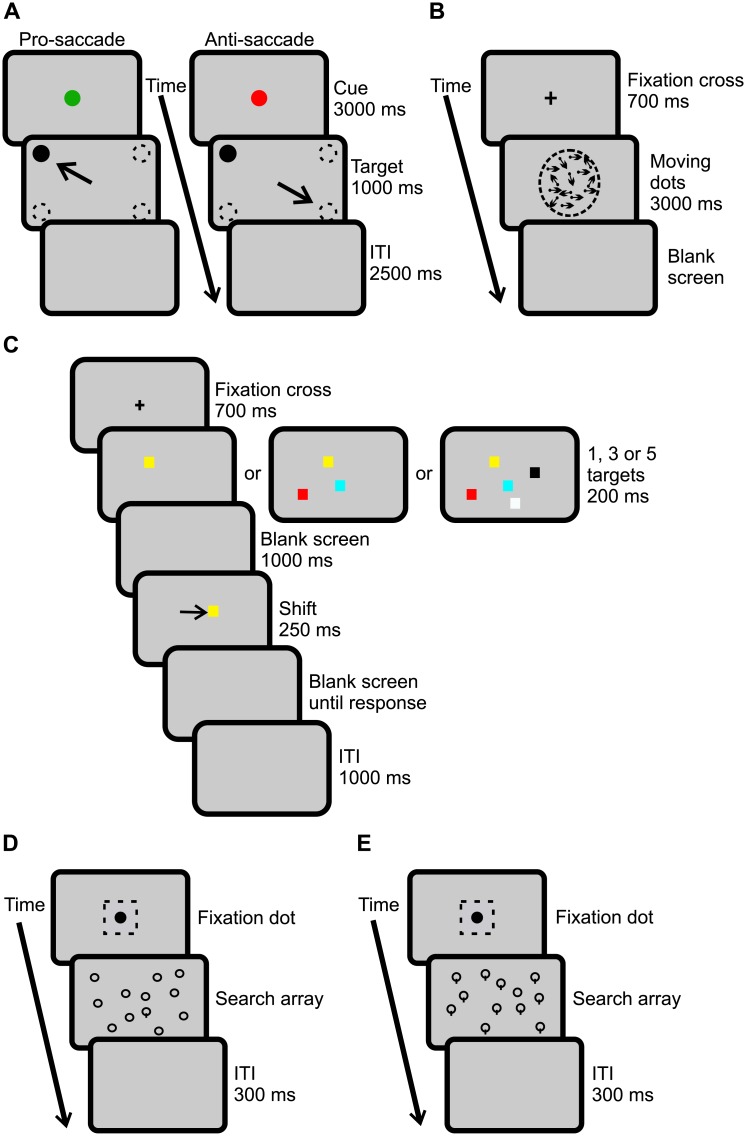
Experimental sequence and timings of the cognitive tasks. (**A**) Interleaved pro/anti-saccade task. In pro-saccade trials (left panel), the cue is green and indicates to participants to make a saccade towards the target. In the anti-saccade condition (right panel), the cue is red, and participants are asked to inhibit a saccade towards the target and to make a saccade 180° away from it instead. The cue remained on the screen for 3000 ms and target appeared for 1000 ms. For both trial types, correct saccades according to cue are illustrated by an arrow while dotted lines represent possible cue locations. There was an inter-trial interval (ITI) was of 2500 ms. (**B**) Decision making task. Participants had to detect the direction, either left or right, of coherently moving dots among randomly moving ones. Each trial began with participants fixating a fixation cross in the center of the screen for 700 ms. Moving dots appeared in a circular window of 10°, illustrated by dotted lines, with a coherence of 10, 20 or 30% among moving dots. The movement of the dots is indicated by arrows in the figure. Following the moving dot screen, a blank screen remained until button-press response and the next trial began. (**C**) Spatial visual memory task. Participants’ instructions were to determine the direction of the shift of one of the targets. Each trial began with a fixation cross for 700 ms in the center of the screen. Next, one, three of five targets appeared for 200 ms. This was followed by a blank screen for 1000 ms, after which one of the targets reappeared for 250 ms but shifted to the right or the left as illustrated by an arrow in the figure. Thereafter the screen remained blank until participant’s button-press response. The ITI lasted 1000 ms. (**D**) Pop-out visual search task. Participants searched for a lollipop shaped target among circles (feature-present) and were asked to report as quickly as possible whether the target was present or absent in the search array. (**E**) Serial visual search task. Targets and distractors were reversed, and participants searched for a circle among lollipops (feature-absent). Trials began with a square fixation window of 4.4° in the center of the screen (illustrated by dotted lines in the figure) surrounding a black fixation dot. When participants’ gaze was detected within this window, the search array appeared and remain until their button-press response. The next trial began after an inter-trial interval (ITI) of 300 ms.

Participants were asked to perform anti- or pro-saccades in response to cues presented on the screen (see Fig 1A for schematised experimental sequence). Each trial began with participants fixating a cue, either a green or red dot (1° diameter), in the centre of the screen for 3000 ms, on a light grey background. The colour of the cue informed participants as to whether they were to perform a pro- (green fixation; left panel in Fig 1A) or anti-saccade (red fixation; right panel Fig 1A). When the fixation cue disappeared, the target (black dot of 1° diameter) appeared at one of the four diagonal locations (8° from centre). In the pro-saccade condition participants gazed at the target, and in the anti-saccade condition participants gazed in the opposite direction (180° from the target). The target remained on the screen for 1000 ms, followed by a blank screen for an inter-trial interval (ITI) of 2500 ms. These timings were set to allow PD patients with bradykinesia (i.e., slow movements) to accomplish the task. Participants were familiarized with the task by a practice block of 10 trials, before beginning the experiment that contained three blocks of 40 trials each. Within each block, 20 trials of each pro- and anti-saccades were interleaved.

#### Decision-making

Participants performed were asked to discern as quickly as possible the direction of the dots moving in a coherent direction among randomly moving ones (Fig 1B). Each trial began with a black fixation cross (1° diameter) presented for 1000 ms on a light grey background. Immediately after the disappearance of the fixation cross, the moving dots were displayed within a 10° circular window in the middle of the screen for 3000 ms. The total number of dots remained constant, but the percentage of coherently moving dots was either 10, 20 or 30, randomized across trials. The direction of the motion was either left or right, also randomized between trials. Participants indicated the direction of the coherently moving dots with one of two button presses. Participants performed 10 practice trials, followed by two experimental blocks of 40 trials each.

#### Spatial visual memory

As depicted in Fig 1C, the participants’ task was to remember the location of one, three or five squares of different colours (black, white, red, green, blue, yellow, or cyan). Each trial began with a black central fixation cross (1° diameter), on a dark grey background, which remained on the screen for 700 ms. Immediately following the extinction of the fixation cross, a randomized number of squares of various colours appeared within a window subtending 5.5–11.5°, for 200 ms. The squares appeared at randomized locations within the window, with a minimal distance of 1.41° from one another. The squares were then replaced by a blank dark grey screen for 1000 ms. Then, one of the squares reappeared for 250 ms but was shifted horizontally, from its original position, by either 0.5°, 2°, or 5° to the left or right, in randomized order [39]. Participants indicated with one of two button presses the direction of the shift. After participants’ response, there was an ITI of 1000 ms during which a dark grey blank screen was displayed. Testing consisted of a practice of 12 trials and three experimental blocks of 54 trials each.

#### Pop-out visual search

Participants were instructed to detect as quickly as possible whether a target was present or absent among distractors within a search array (Fig 1D). Each trial began with a black fixation dot (1° diameter) set against a light grey background. A gaze verification was applied within a window of 4.4*4.4° around the fixation dot. Once gaze was detected within this window, the fixation dot was replaced with a rectangular search array (25*18°), which remained on the screen until participants responded by one of two button presses. The search array was randomly composed of either 12, 24 or 48 items, including distractors and the target, if present. Distractors consisted of circles (1.1° diameter), while the target, when present, was the same circle except with a vertical line (0.75°) crossing the bottom half, essentially resembling a lollipop (also known as a feature-present visual search) [45]. Once participants responded, the search array was replaced by a blank screen for an ITI of 300 ms. Testing began with 12 practice trials, followed by one block of 81 trials of which 72 were target-present (24 repetitions of each condition—12, 24, or 48 items) and 9 were target-absent trials, in a randomized order.

#### Serial visual search

The instruction and protocol of the task were the same as for the pop-out visual search described above. However, target and distractors were reversed: participants were asked to search for the plain circle among lollipops (Fig 1E, known as feature-absent visual search) [45]. While bottom-up information processing can be measured with the pop-out task, the serial tasks solicit top-down processes [37,38]. The number of trials for the practice session and the experimental block, as well as the other parameters (the three conditions, and the ratio of present/absent trials) were the same as described above in the pop-out visual search.

### Data analysis

#### Interleaved pro/anti-saccade task

After the analysis of recording trials, it was revealed that 11 participants (five younger adults, one older adult and five PD patients) made an eye-movement 90° away from target during anti-saccade trials instead of 180 ° away. We concluded these participants did not understand the task instructions and they were thus excluded from subsequent analyses. From the remaining participants, we recorded 5,136 trials, from which we first removed trials where the saccade reaction times (SRTs) were less than 100 ms or more than 1000 ms (28 trials, 0.5% of total trials). Next, we removed trials where the saccades had start positions above 5 degrees from the centre of the screen (14 trials, 0.3% of total trials) or extreme end position (12 trials, 0.2% of total trials). We also removed amplitude outliers, defined as trials where the saccades were smaller than 2° or larger than 20° (91 trials, 1.8% of total trials). Finally, we removed all trials with a saccade amplitude beyond three standard-deviations (SDs) from each participant’s mean saccade amplitude (52 trials, 1% of total trials). There remained 4,939 trials (96.2% of total trials).

We conducted our statistical analyses on error rates (ERs) and SRTs for pro- and anti-saccade trials. For ERs, we defined incorrect pro-saccades as saccades away from the target, while incorrect anti-saccades were saccades to the target. Separately for pro- and anti-saccades, we then divided the number of incorrect trials by the total number of trials per participant. SRTs were calculated only from correct trials, by taking the mean saccade reaction time for each participant, for each condition (pro- and anti-saccade).

#### Decision-making

We defined decision times (DT) as participants’ response time on correct trials. We conducted an outlier analysis per participant on their DT collapsed across coherence levels, from a total of 5,956 trials. We also removed outliers as trials with DT longer than three SDs of each participant’s mean (112 trials, 1.9% of total trials). There remained 5,844 trials (98.1% of total trials).

#### Spatial visual memory task

We collected a total of 10,808 trials, all of which were used in the analyses. Percentage correct was defined as the number of correct trials divided by the total number of trials, collapsed across the number of squares.

#### Pop-out and serial visual search tasks

We calculated participants’ search times (STs) separately for each visual search task as follows: the time between the onset of the search array and participants’ button-press, only including correct trials. From a total of 12,068 recorded trials, we first removed extreme STs for each visual search task, i.e., any ST longer than 9000 ms (24 trials, 0.002% of total trials). Second, we removed ST outliers on an individual basis: these were calculated per participant and per type of visual search as two SDs outside their mean ST, or 95% of each participant’s overall trials (734 trials, 6.1% of total trials). For statistical analyses, there remained 11,310 trials (93.7% of total trials) with which we calculated the mean STs collapsed across item number (12, 24, and 48) for each visual search task.

Target absent trials were not analysed but were included as catch trials to ensure that participants were performing the task correctly, i.e. pressing the button when they detected the button rather than automatically pressing the button at every trial. To confirm that participants were following instructions, we compared performance for target present and absent trials by calculating ERs and performing a mixed ANOVA with group and target (present and absent) as factors.

For each of the tests listed above, we compared performance across groups with ANOVAs and followed up any significant results with the appropriate post-hoc tests. Full data set is available in S1 File.

## Results

We began our statistical analyses by comparing performance in the interleaved pro/anti-saccade, decision-making, visual search and spatial visual memory tasks across groups. This provided an overview of each group’s baseline performance. We followed these analyses by conducting preliminary correlations to explore relationships between task performance, specifically how anti-saccade performance related to the other tasks. We lastly performed regression analyses for PD patients and older adults to investigate how anti-saccade performance predicted performance in the other tasks.

### Interleaved pro/anti-saccade task: Error rates (ERs), saccade reaction times (SRTs)

**Fig 2:**
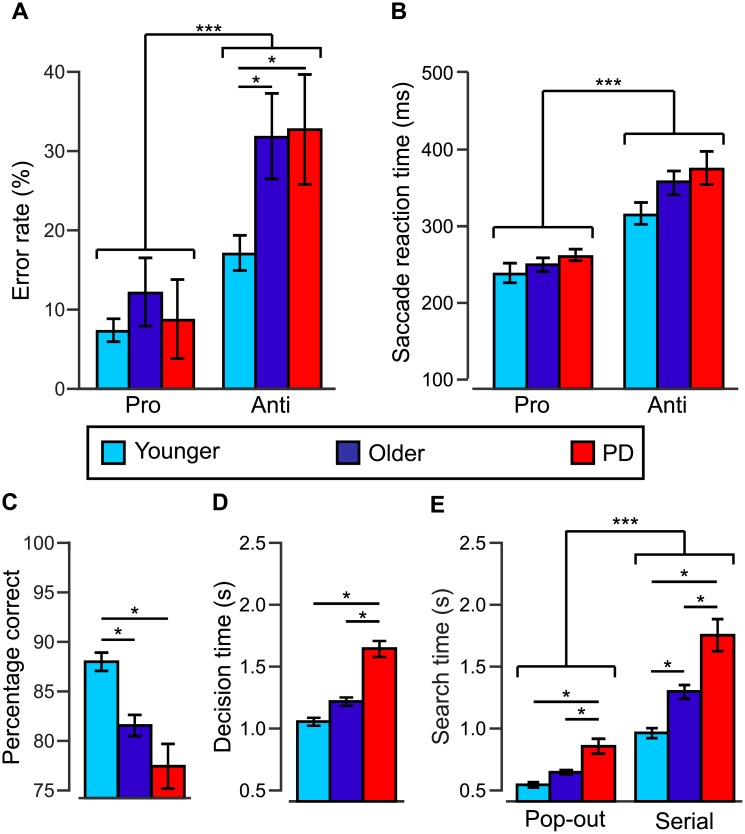
Performance across groups in the saccade and cognitive tasks. Younger adults’ performance is shown in blue, older adults’ in purple and PD patients’ in red. (**A**) Mean error rates in percentages for pro-saccades and anti-saccades. Pro, pro-saccade trials; Anti, anti-saccade trials. (**B**) Mean saccade reaction times in ms for each group. (**C**) Decision times for correct decisions in the decision-making task. (**D**) Performance in the spatial visual memory task in percentage correct. (**E**) Search times both the pop-out and serial visual search tasks. We compared group performance in each panel with one-way ANOVAs and corrected t-tests, * = p<0.05, *** = <0.001. Bars represent standard error of the mean across subjects for each group.

We compared pro- and anti-saccade ERs, and pro- and anti- SRTs to validate of our interleaved pro/anti-saccade paradigm for each population tested (Fig 2A). As expected, repeated measures ANOVAs revealed that pro-saccade ERs were significantly lower than anti-saccade ERs, *F*(1, 64) = 53.44, *p* < .001, and that pro SRTs were also significantly lower than anti SRTs, *F*(1, 64) = 1198.86, *p* < .001. These results replicate previously reported patterns [15,38] and is therefore valid.

We next assessed differences across groups for ERs and SRTs. One-way ANOVAs for pro-saccade ERs, *F*(2, 64) = .45, *p* = .64, and SRTs, *F*(2,64) = .34, *p* = .71, did not reach significance. In contrast, for anti-saccade ERs, there was a significant main effect of group, *F*(2, 64) = 4.16, *p* = .02. Older adults (*M* = 32.00%, *SD* = 24.71%) and PD patients (*M* = 30.49%, *SD* = 26.37%) had significantly higher ERs than younger adults (*M* = 16.68%, *SD* = 11.96%), *t*(48) = 15.675, *p* = .005; *t*(42) = 13.34, *p* = .02, while there was no significant difference between PD patients and older adults, *t*(34) = -.18, *p* = .86 (Fig 2A). As for anti SRTs, there was no main effect of group, *F*(2, 64) = 2.11, *p* = .13 (Fig 2B). However, there is a tendency for SRTs to increase with both age and pathology. The lack of effect found here thus appears to be due to lack of statistical power as many studies have showed increased SRTs in older adults [46] and PD patients [20,47].

#### Decision-making task: Decision time (DT)

For decision-making times, we also found a significant main effect of group, *F*(2, 74) = 7.554, *p* = .001. Results showed that PD patients had longer DTs (M = 1674.25 ms, SD = 766.09 ms) than younger adults (*M* = 1093 ms, *SD* = 376.98 ms) and older adult (*M* = 1238.36 s, *SD* = 473.59 ms; *t*(51) = -3.691, *p* = .001; *t*(40) = 2.24, *p* = .03, respectively. Further, there was no significant difference between older and younger adults’ DTs, *t*(53) = -1.26, *p* = .21 (Fig 2C). This suggests that decision-making abilities, as measured by our coherent motion detection task, tend to decrease with aging and appear to be further delayed by PD.

### Spatial visual memory task: Percentage correct

For the spatial visual memory task, we found a significant main effect of group on performance, *F*(2, 75) = 12,164, *p* < .001. Indeed, younger adults (*M* = 88.17%, *SD* = 5.20%) performed significantly better than older adults (*M* = 81.78%, *SD* = 5.02%) and PD patients (*M* = 77.68%, *SD* = 10.08%; *t*(54) = 4.555, *p* = .001; *t*(52) = 5.052, *p* < .001, respectively), while performance between older adults and PD patients was not significantly different, *t*(40) = -1.691, *p* = .099 (Fig 2D). These results show a decline in spatial visual memory performance with age.

### Visual search tasks: Error rates (ERs), and search time (ST)

We first confirmed that participants performed the task according to given instructions. We conducted statistical analyses as outlined in the Method section for error rates and we found no main effect of group or target and no interaction effect for pop-out, *F*(2, 69) = 2.40, *p* = 0.92; *F*(1, 69) = 1.47, *p* = 0.23; *F*(2, 69) = 1.83, *p* = 0.17, and serial search, *F*(2,71) = .12, *p* = .88; *F*(1,71) = .14, *p* = .71; *F*(2,71) = .48, *p* = .62. Further, these results illustrate similar accuracy across groups for both visual search tasks.

Mean STs are plotted in Fig 2E. They followed the expected pattern; in both pop-out and serial tasks young adults had the lowest STs, and PD patients had the highest STs. For pop-out search, we observed a significant main effect of group, *F*(2, 71) = 22.489, *p* < .001. Pop-out STs for PD patients (*M* = 945.08 ms, *SD* = 257.08 ms) were significantly longer than younger (*M* = 633.40 ms, *SD* = 123.66 ms) and older adults’ (*M* = 735.71 ms, *SD* = 78.79 ms; *t*(49) = -5.863, *p* < .001; *t*(37) = 3.549, *p* < .001). In contrast, the difference between the two control groups’ STs only showed a trend, *t*(52) = -3.38, *p* = .07. For serial search, we also found a significant main effect of group, *F*(2,73) = 35.05, *p* < .001; in this case all three groups were different from each other. PD patients’ STs (*M* = 1776.89 ms, *SD* = 437.51) were higher than younger adults’ (*M* = 1052.98 ms, *SD* = 235.07 ms), and older adults’ (*M* = 1389.22 ms, *SD* = 254.04 ms), and older adults had longer ST than younger adults, *t*(51) = -7.86, *p* < .001; *t*(38) = 3.47, *p* < .001; *t*(53) = -5.00, *p* < .001. Overall, these results suggest PD patients have significantly impaired performance in visual search types invariably of task type compared to older adults. In addition, the latter cohort show deficits in the serial search task compared to younger adults.

### Regression analysis

**Fig 3:**
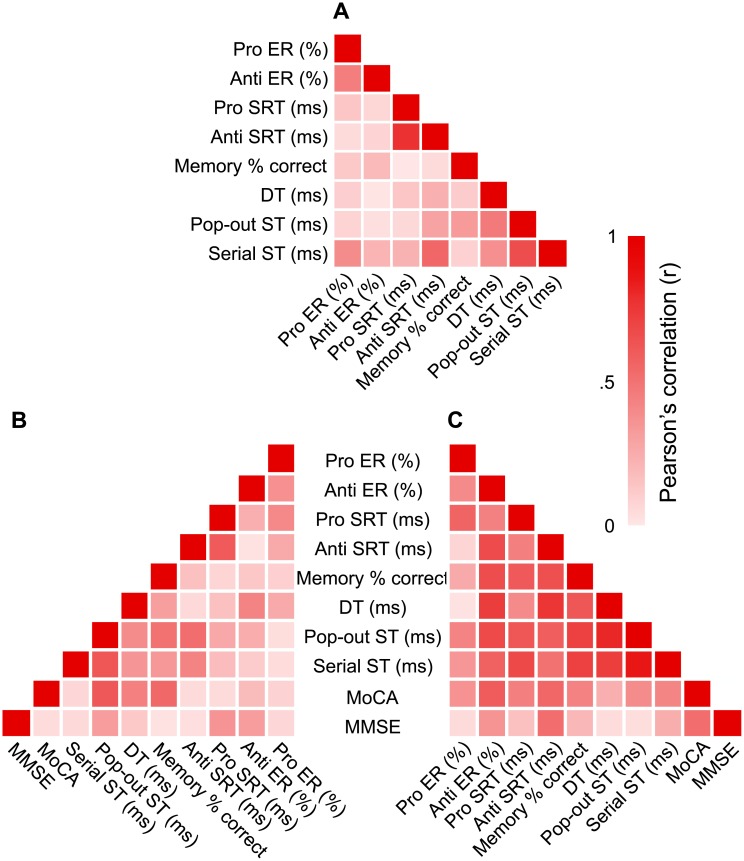
Heat maps of correlations among all tasks for each group. Pearson’ correlations between the performance measures for the different tasks for younger adults in (**A**), older adults in (**B**), and PD patients in (**C**). Weak correlations, near 0, are in white while those nearing 1 are in red, portraying strong correlations. Pearson’s correlations are in absolute values.

We first performed correlational analysis between all measures of our tasks to determine whether there were any relationships between participants’ performance in the interleaved pro/anti-saccade task and their performance in the other cognitive tasks. These preliminary correlational analyses (Fig 3) demonstrates that while performance in the cognitive tasks and the interleaved pro/anti-saccade task were overall not correlated in young adults (Fig 3A), they were often correlated in older adults (Fig 3B), and were particularly strongly correlated in PD patients (Fig 3C). In this figure, weak correlations are illustrated in white and strong ones, in red. The overall weak correlations in young adults is likely due to ceiling effects—the tasks were, for the most part, too easy for the young adults. For this reason, we removed young adult data from further regression analyses, but these results are available in S1 Table. We also did not consider pro SRTs and pro ERs in the regression model; because of the automatic nature of pro-saccades, they were only a means to induce task-switching in participants. Thus, we only included measures obtained from the anti-saccade task in a two-level hierarchal linear regression model to distinguish between correlation strength and group effects for each task: we first tested anti SRTs and anti-saccade ERs as predictors (level 1); we then assessed for an effect of group (level 2) as an additional predictor (i.e., a difference between older adults and PD patients). We determined whether performance in anti-saccades predicted performance of the spatial visual memory, decision-making, pop-out visual search, and serial visual search tasks, and scores on the MoCA and MMSE (see Table 3).

**Table 3:** Standard multiple linear regression of anti-saccade measures on cognitive task performance for older populations.

Tasks	Model	Predictors	Unstandardized coefficients		Standardized coefficients		*R*^*2*^	R^2^ change	*F*	*p*
			*B*	*SE*	ß	*p*				
Memory	1						.349	.349	8.863	.001
		Anti SRT	-.022	0.013	-.310	.096				
		Anti ER	-.093	0.049	-.344	.066				
	2						.359	.010	0.478	.494
		Anti SRT	-.021	0.013	-.292	.122				
		Anti ER	-.096	0.049	-.358	.060				
		Group	1.333	1.928	.099	.494				
DM	1						.459	.459	14.015	< .001
		Anti SRT	1.939	.824	.388	.025				
		Anti ER	6.834	3.115	.362	.035				
	2						.472	.013	.764	.389
		Anti SRT	1.836	.835	.368	.035				
		Anti ER	7.144	3.146	.378	.030				
		Group	-107.318	122.778	-.113	.389				
Pop-out	1						.296	.296	6.530	.004
		Anti SRT	.663	.269	.456	.020				
		Anti ER	.754	1.043	.134	.476				
	2						.455	.159	8.765	.006
		Anti SRT	.578	.243	.397	.024				
		Anti ER	.903	.935	.160	.341				
		Group	-106.441	35.954	-.402	.006				
Serial	1						.175	.175	3.389	.046
		Anti SRT	1.234	.645	.377	.065				
		Anti ER	.831	2.545	.064	.746				
	2						.280	.105	4.535	.041
		Anti SRT	1.032	.620	.316	.106				
		Anti ER	1.159	2.420	.090	.635				
		Group	-195.529	91.820	-.329	.041				
MoCA	1						.146	.146	2.815	.074
		Anti SRT	-.008	.005	-.299	.159				
		Anti ER	-.011	.020	-.114	.585				
	2						.301	.155	7.120	.012
		Anti SRT	-.006	.005	-.227	.247				
		Anti ER	-.016	.018	-.172	.376				
		Group	1.899	.712	.398	.012				

*N* = 35. DM, decision-making; SE, standard error; MoCA, Montreal Cognitive Assessment.

We found that performance in the anti-saccade task significantly predicted performance in the decision-making task. With SRT and ER as predictors, 46% of the variability in decision time was explained, *F*(2, 33) = 14.02, *p* < .001. Considering the large amount of the variance explained, anti-saccade measures are good predictors at the group-level for both tested populations. Adding Group as a predictor did not contribute significantly to the model’s fits (*R*^*2*^_Δ_ = .47, *F*_Δ_(1, 32) = 0.76, *p*_Δ_ = .39). This suggests increases in SRTs and ERs are related to increases of DT in both groups. In other terms, participants who took longer to perform anti-saccades, and made more anti-saccade errors, had increased DT in the decision-making task.

Similarly, performance on the spatial visual memory task was significantly predicted by performance on the anti-saccade task. The anti SRT and ER predicted 35% of the variability in the proportion of trials were the position of the squares were correctly remembered, *F*(2, 33) = 8.86, *p* = .001. Nonetheless, the variance explained for this task was moderate, suggesting that the unexplained variance might be due to individual differences such as MoCA scores, age, medication intake and disease progression (i.e., Hoehn & Yahr stages) in the specific case of the PD group. Adding Group did not significantly improve the model (*R*^*2*^_Δ_ = .36, *F*_Δ_(1, 32) = 0.48, *p*_Δ_ = .49). Overall, the relationship among the variables and predictors show that increases in SRT and ER are related to decreases in percentage correct. This suggests that participants who had longer SRTs and higher ERs, were more likely to make more mistakes on the spatial visual memory task.

In contrast, the two visual search tasks required different regression models than those described for predicting performance in the spatial visual memory and decision-making tasks. Specifically, the anti-saccade predictors in the first level of the model explained 27% of variation in ST for the pop-out task, *F*(2, 31) = 6.53, *p* = .004, and adding group significantly improved the model, *F*_Δ_(1, 30) = 8.77, *p*_Δ_ = .006. Taken together, predictors explained 46% of variation of ST for the pop-out task, which supports the idea that the reported relationship was related to inter and intra-group factors. The predictive strength of the combined anti-saccade factors (ß = .56) was similar to the strength of the group effect (ß = -.40). Group was a significant predictor because pop-out STs were strongly correlated with anti-saccade performance in PD, but to a much lesser extent, if at all, in older adults (see correlations in Fig 3B and 3C). As anti-saccade performance improved (i.e., ERs and SRTs decrease), pop-out STs decreased in PD patients to a larger extent than in older adults.

The serial visual search showed similar results as for pop-out visual search: the first level determined that anti-saccade performance significantly predicted ST (*R*^*2*^ = .18, *F*(2, 32) = 3.39, *p* = .046) and the addition of group as a factor in the second level significantly improved the model’s fit (*R*^*2*^_Δ_ = .21, *F*_Δ_(1, 31) = 4.54, *p*_Δ_ = .041). Nonetheless, the variance explained remained low in this second model’s fits; the large amount of unexplained variance is likely due to individual differences, such as age or characteristics related to PD. Once more considering correlational relationships, we observed a strong significant correlation between anti-saccade ER and serial ST in PD patients, but not in older adults (Fig 3B and 3C). As shown for the pop-out visual search task, the predictive strength of the combined anti-saccade factors (ß = .41) was similar to the strength of the group effect (ß = -.33). Increases in anti SRTs and ERs are related to increase in serial STs in PD patients and less so, in older adults. In summary, the significant effect of group demonstrates that performance in the anti-saccade trials is a stronger predictor of performance in the visual search tasks in PD patients, compared to older adults.

Lastly, we attempted to predict MoCA and MMSE scores from anti-saccade performance. For the first test, anti-saccade performance marginally significantly predicted the variation in MoCA scores (*R*^*2*^ = 0.15, *F*(2, 33) = 2.815, *p* = .074) and the addition of group as a predictor improved the model, so that a total of 30% of variation in MOCA scores were explained, *F*_Δ_(1, 32) = 7.120, *p*_Δ_ = .012. The extent variance explained does not exclude individual differences within each group, for example age and characteristics related to PD. However, the majority of the significant effects were driven by the strong correlations between both anti-saccade performances and MoCA scores in PD patients, which were not observed in older adults (Fig 3B and 3C). This is explained by similarities found between the strength of combined anti-saccade factors (ß = -.40) and the strength of the group factor (ß = -.40). As anti-saccade performance increased, MoCA scores decreased, but more so in PD patients than in older adults.

Nevertheless, anti-saccade performance did not significantly predict MMSE scores (*R*^*2*^ = .027, *F*(2, 33) = 0.429, *p* = .636), and the addition of group did not lead to an improvement in the regression model (*R*^*2*^_Δ_ = .074, *F*_Δ_(1, 32) = 1.637, *p*_Δ_ = .210). In sum, while anti-saccade performance was related to MOCA scores, particularly in PD patients, we found no such relationship to MMSE scores.

## Discussion

In the present article, we aimed to predict cognitive functioning in three subject groups (young healthy adults, older healthy adults, and PD patients) with a saccade task known to measure cognitive control. We initially showed that young adults had the best performance in all of our cognitive tasks—in fact they often performed at ceiling. In contrast, PD patients were impaired in decision-making and visual search tasks compared to their age-matched controls. Aging itself tended to degrade performance across all tasks. Additionally, regression analyses revealed that the measures of anti-saccade performance—ERs and SRTs—could predict performance in spatial visual memory and decision-making tasks in older adults and PD patients. In the specific cases of visual search and MoCA, performance in anti-saccades were more predictive for PD patients compared to healthy older adults. Taken together, our results support the hypothesis that anti-saccade performance reflects cognitive abilities and therefore can provide generalized measures of cognitive control.

Cognitive decline in normal aging has been attributed to frontal lobe degeneration [48,49]. Recent studies have shown that dysregulation in the parieto-frontal and striato-frontal pathways can also cause deficits in cognitive functioning. The role of the parietal remains ambiguous in inhibitory mechanisms related to the anti-saccade task [19,50–58]. However, it has been shown but PD inhibitory control deficits may be explained with over-activation of this structure to compensate for dopamine loss in the basal ganglia [59]. As for dysregulations in the striato-frontal pathways, they are associated with deficits in cognitive control in both PD and normal healthy aging [60,61]. For example, visuospatial memory relies on the integrity of the dorsolateral prefrontal cortex [62,63], the parietal cortex [64], the caudate [62,65] and the mediodorsal thalamic nucleus [66]; all regions included in the striato-frontal or parieto-frontal pathways [67–69]. The striatum and the dorsolateral prefrontal cortex are also critical for the speed-accuracy trade-off underlying our decision-making task [70,71]. The fact that anti-saccades also recruit the striato-frontal and parieto-frontal neural substrates explains our reported correlations between anti-saccade performance and spatial visual memory, and decision-making abilities [19,27,72]. Further, it may suggest these substrates mediate the relationship between cognitive control and decision-making and visual memory processes.

Both the frontal-striatum and the parieto-frontal neural networks are crucial for inhibitory control and task-switching abilities underlying most measures of cognitive control [18,73–75]. The role of the prefrontal cortex in anti-saccade inhibitory mechanisms has been shown in multiple neurophysiological studies. For example, its cortical activity predicts the level of performance in memory-guided and anti-saccade tasks [54]. Other studies have demonstrated increased pre-stimulus activity during anti-saccade trials [76,77] as well as changes in saccade triggering thresholds [78], reflecting pre-emptive top-down inhibitory mechanisms [55]. Neuroimaging studies have also revealed the specific involvement of the frontal cortex in the inhibition of responses [79]. Similarly, task-switching has implicated both the prefrontal and parietal networks [80–82]. Given the recruitment of both inhibitory and task-switching abilities, anti-saccade tasks can be effective in neuropsychological settings as a measure of function within these networks [55].

Although most neuropsychological standardized tests, such as the Stroop task, are often developed with the intent of measuring one specific cognitive function, studies show that they tend to measure more than one cognitive ability [83–85] and to correlate with other non-related standardized tasks [86]. This is likely because most standardized tests implicate similar cognitive abilities, and most importantly cognitive control. Thus, the interleaved pro/anti-saccade task presents the advantage of measuring cognitive control through individuals’ inhibitory control and task-switching functioning.

We observed a difference between PD patients and older adults in the ability of anti-saccades to predict performance in some of the cognitive tasks measured here (e.g. visual search and MoCA); the predictive pattern of anti-saccades for the visual search tasks differed across groups unlike from the ones observed for the spatial visual memory and decision-making tasks. Decreases in visual search tasks’ performance are observed in abnormal aging [87–89], which could suggest it requires more damage to the cortex before detection of cognitive impairment is possible via these tasks. In our study, PD patients were more cognitively impaired and more variable in their performance than the older adult population. As we noted stronger correlations in the linear fits of anti-saccade performance and search times in both visual search tasks in PD patients, compared to older adults, this may merely reflect a general impairment in information processing associated with the disorder [90]. Indeed, older adults have less of an impairment in visual information processing compared to PD patients [91] and in attention [2], both abilities inherent to the performance of visual search tasks [92]. Therefore, older adults would be less delayed in their performance on the visual tasks compared to PD patients.

We also reported more severe cognitive impairment in PD patients, as revealed by significantly lower MoCA scores. This difference could explain the stronger relationship between MoCA scores and anti-saccade measures in PD patients compared to older adults. In contrast, there was no significant relationships between MMSE and anti-saccade performance, which is likely due to the lack of variability in MMSE scores of participants included in the study. MMSE was designed as a tool to assess severe cognitive impairment, and is specific and sensitive only to such sever impairments [41], while MOCA scores are more sensitive to mild cognitive impairments. In our study, moderate-to-severe cognitive impairment was an exclusion criterion, because of the complexity of the anti-saccade task. Therefore, we demonstrated that the anti-saccade task was related to mild cognitive impairment as measured by the MoCA, but not to MMSE scores.

It is important to note that all our participants were medicated, in most cases with dopamine agonists (see Table 1). We did not control for medication intake and dosage in this first exploratory study, for ethical reasons. Dopamine circuits are important in the regulation of striato-frontal pathways [93,94]. Further, dopaminergic medications such as Levodopa has been shown to decrease anti-saccade errors in PD patients [95] and its withdrawal is associated with impaired task-switching abilities [96]. Thus, medication could have influenced our findings. Further studies are needed to directly explore the effects of dopamine on cognition with carefully controlled medications.

We propose that the interleaved pro/anti saccade task may also have potential as a cognitive rehabilitation tool for clinical populations. Anti-saccade training is effective in younger [97] and older adults [98], and clinical populations [99,100]. Due to the relationships between anti-saccade performance and performance in other cognitive tasks reported here, training in our saccade task may improve connectivity in the striato-frontal and parieto-frontal pathways, and, in turn, increase inhibiting and task-switching abilities. Thus, anti-saccade training may transfer to improvement in a wide variety of cognitive functions in older adults and PD patients.

In conclusion, we demonstrated that performance in the interleaved pro/anti-saccade task is related to decline in cognitive functions as measured by spatial visual memory, decision-making, visual search, and MoCA. We suggest that the dysregulation of the striato-frontal and the parieto-frontal neural pathways likely underpin this relationship. Our findings demonstrate the sensitivity of the anti-saccade task as a cognitive marker for cognitive function in older healthy and pathological populations. Due to the relative simplicity of the anti-saccade task, it would be a particularly useful complementary task for neuropsychological testing in mild cognitive impaired populations, even in medicated populations (such as our PD patients).

## Supporting information

S1 Table Standard multiple linear regression of anti-saccade measures on cognitive task performance for younger adults. *N* = 30. DM, decision-making; SE, standard error.
(DOCX)

S1 File Participant dataset with mean performance. Data shows mean per participant. Missing or non-applicable data is indicated by a dot. Anti, anti-saccade; DT, decision time, ER, error rate; MoCA, Montreal cognitive assessment; MMSE, Mini- Mental State examination; ms; milliseconds; pro, pro-saccade; SRT, saccade reaction time; ST, search time; y, year.
(XLSX)
