# Supplementary material for: Anti-saccades predict cognitive functions in older adults and patients with Parkinson’s disease
Source: PLoS One. 2018 Nov 28;13(11):e0207589. doi: 10.1371/journal.pone.0207589 (PMC6261587; doi:10.1371/journal.pone.0207589)
Supplement: S1 Table — N = 30. DM, decision-making; SE, standard error. (DOCX) [file pone.0207589.s001.docx]

**Table S1. Standard multiple linear regression of anti-saccade measures on cognitive task performance for younger adults**

| Tasks | Predictors | Unstandardized  coefficients | | Standardized  coefficients | | *R^2^* | *F* | *p* |
| --- | --- | --- | --- | --- | --- | --- | --- | --- |
|  |  | *B* | *SE* | ß | *p* |  |  |  |
| Memory |  |  |  |  |  | .038 | .512 | .605 |
|  | Anti SRT | .004 | 0.012 | .063 | .747 |  |  |  |
|  | Anti ER | -.074 | 0.075 | -.189 | .336 |  |  |  |
| DM |  |  |  |  |  | .053 | .693 | .509 |
|  | Anti SRT | 1.099 | .934 | .229 | .250 |  |  |  |
|  | Anti ER | -.456 | 5.757 | -.015 | .938 |  |  |  |
| Pop-out |  |  |  |  |  | .084 | 1.149 | .333 |
|  | Anti SRT | .447 | .316 | .290 | .144 |  |  |  |
|  | Anti ER | .044 | 1.957 | .004 | .982 |  |  |  |
| Serial |  |  |  |  |  | .328 | 6.354 | .006 |
|  | Anti SRT | 1.796 | .542 | .534 | .003 |  |  |  |
|  | Anti ER | 3.490 | 3.312 | .170 | .302 |  |  |  |
